# Unraveling the Pleiotropic Role of High-Density Lipoproteins (HDLs) in Autoimmune Rheumatic Diseases

**DOI:** 10.1155/2024/1896817

**Published:** 2024-11-14

**Authors:** Marcia B. Giacaglia, Vitória Pires, Monique F. M. Santana, Marisa Passarelli

**Affiliations:** ^1^Programa de Pós-Graduação em Medicina, Universidade Nove de Julho (UNINOVE) 01525-000, São Paulo, Brazil; ^2^Laboratório de Lípides (LIM10), Hospital das Clínicas (HCFMUSP) da Faculdade de Medicina da Universidade de São Paulo 01246-000, São Paulo, Brazil

## Abstract

Autoimmune rheumatic diseases (ARDs) exhibit an elevated incidence of cardiovascular disease (CVD). The elevation of inflammatory and immune stress accompanying ARDs contributes to atherosclerosis development and alterations in lipid metabolism and lipoprotein profile add to cardiovascular (CV) risk. The plasma concentration of high-density lipoprotein cholesterol (HDLc) is inversely related to CVD and serves as a discriminator of CV risk. However, this association is not unequivocal, and changes in HDL functionality appear to emerge as a better indicator of CV risk, albeit difficult to measure and monitor clinically. The modulation of HDLc itself can bring benefits in controlling autoimmunity and reducing ARD activity. Understanding HDL function and each peculiarity involved in ARDs enables to seek means to prevent ischemic outcomes associated with CVD, in the face of the residual CV risk persisting even with controlled disease activity and classic risk factors. By comprehending HDL's structural and functional nuances, it will be possible to develop more effective strategies to manage the evolution and outcomes of ARDs. It is also necessary to standardize diagnostic methods and establish different markers for each specific disease allowing the design of intervention strategies to restore HDL functionality, reduce residual CV, and prevent, alleviate, or even suppress ARD activity.

## 1. Introduction

Autoimmune rheumatic diseases (ARDs) encompass a wide range of clinical and laboratory manifestations, characterized by varying degrees of systemic immune responses and chronic inflammation. Episodes of exacerbation alternate with periods of remission, associated with metabolic alterations that contribute to the overall pathophysiological profile. The triggers and pathophysiology of these disorders are not fully understood, but many aspects of their pathogenesis are being elucidated, enabling the development of more specific treatments.

ARDs encompass several distinct diseases, such as rheumatoid arthritis (RA), systemic lupus erythematosus (SLE), psoriatic arthritis (PsA), inflammatory spondyloarthropathies (SpA), and Sjögren's syndrome (SS). These diseases share similar clinical, laboratory, and immunological manifestations. The etiology of this group of diseases is still unknown, and the complex pathogenic mechanism has only been partially elucidated. In the development of ARDs, there is an intricate action of environmental factors, epigenetic modification, and genetic susceptibility, which trigger the autoimmune reaction [1]. It is estimated that 1.3 million North American adults are impacted by RA, 0.6 million to 2.4 million by SpA, 161,000–322,000 by SLE, and 0.4 million to 3.1 million by SS [2].

**Figure 1:**
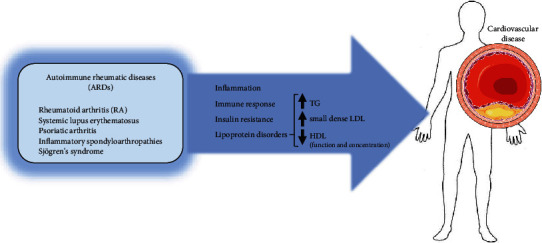
Autoimmune rheumatic diseases and atherosclerosis. Autoimmune rheumatic diseases (ARDs), including rheumatoid arthritis, systemic lupus erythematosus, psoriatic arthritis, inflammatory spondyloarthropathies, and Sjögren's syndrome, are associated with an elevated risk of cardiovascular disease. This is primarily driven by chronic inflammation, an exacerbated autoimmune response, insulin resistance, and alterations in plasma lipid profiles. These lipid abnormalities are characterized by increased plasma triglycerides (TGs), the formation of small, dense LDL particles, reduced HDL cholesterol levels, and impaired HDL particle functionality (parts of the figure were obtained from https://smart.servier.com).

Individuals with ARDs face an elevated risk of atherosclerosis, attributed not only to inflammatory and immunological stress but also to metabolic shifts that promote a proatherogenic environment. Insulin resistance, which develops alongside low-grade chronic inflammation, leads to changes in lipid and lipoprotein metabolism, marked by elevated plasma levels of triglycerides (TGs), small dense atherogenic low-density lipoproteins (sdLDL), and diminished high-density lipoprotein cholesterol (HDLc) (Figure 1). Interestingly, the control of classic risk factors and clinical management of the disease does not fully eliminate the risk of cardiovascular (CV) morbidity and mortality, and residual risk is attributed to the presence of nonclassic risk factors. These include the accumulation of oxidation and glycoxidation products that exacerbate local and systemic inflammatory insults. Furthermore, they are represented by functional alterations in lipoprotein particles, namely, HDL which may exhibit a reduction in their ability to counteract the formation of atherosclerotic lesions [3–7].

## 2. High-Density Lipoproteins (HDLs)

HDLs exhibit heterogeneity in both size and composition ranging from 7.6 to 10.6 nm in diameter and consisting of approximately 50% apolipoproteins (apos), 20% total cholesterol, 15% phospholipids (PLs), and 5% TG. Among these components, apoA-1 stands out as the primary protein, playing a pivotal role in most functions attributed to HDL. HDLs are formed as lamellar apoA-1/PL-containing structures called prebeta HDL produced by the liver and intestine, and from the detachment of surface components of TG-rich lipoproteins (very low-density lipoproteins (VLDLs) and chylomicrons (QMs)) during lipolysis by lipoprotein lipase (LPL). HDL generation relies on insulin signaling, making it susceptible to compromise in the presence of insulin resistance. In this state, the establishment of hypertriglyceridemia is linked to the reduction of HDLc.

**Figure 2:**
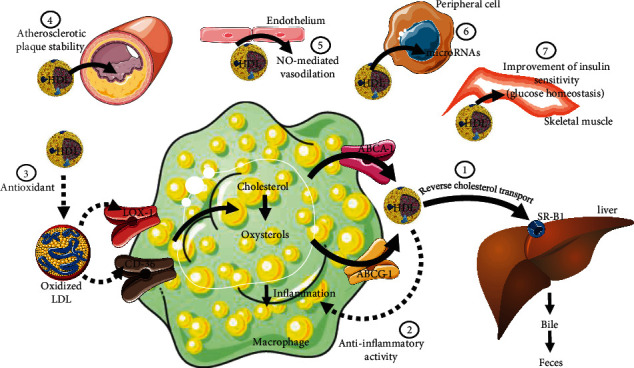
High-density lipoprotein (HDL) functionality. HDL performs various functions that cannot be inferred solely by measuring HDL cholesterol (HDLc) or apolipoprotein A-1. These functions link HDL to cardiovascular protection and its role in the pathophysiology of several chronic and acute diseases. (1) HDL mediates reverse cholesterol transport, removing excess cellular cholesterol and oxysterols (including from macrophages infiltrated in the arterial intima) and promoting its trafficking to the liver for elimination via bile and feces; (2) diminishes the inflammatory response by minimizing the transcription of inflammatory genes; (3) reduces LDL oxidation, decreasing its uptake by macrophages; (4) stabilizes atherosclerotic lesions by carrying protease inhibitors; (5) promotes vasodilation by enhancing nitric oxide (NO) release from the endothelium; (6) transports microRNAs and delivers them to target cells, regulating protein expression; and (7) supports glycemic homeostasis by positively modulating insulin sensitivity. These functions may be compromised in ARDs contributing to CDV. Abbreviations: ABCA-1, ATP-binding cassette transporter A-1; ABCG-1, ATP-binding cassette transporter G-1; CD-36, cluster of differentiation 36—member of class B scavenger receptor; LOX-1, lectin-type oxidized low-density lipoprotein (LDL) receptor-1; SR-B1, scavenger receptor class B type 1 (parts of the figure were obtained from https://smart.servier.com).

By acquiring free cholesterol from peripheral cells, HDL enriches its core with esterified cholesterol (EC) due to the activity of lecithin cholesterol acyltransferase (LCAT). Additionally, through the action of the cholesteryl ester transfer protein (CETP), HDL receives TG from TG-rich lipoproteins, gradually increasing in size and maturation to facilitate cholesterol delivery to the liver and its secretion into bile and excretion in feces. This metabolic process is part of the reverse cholesterol transport (RCT), an antiatherogenic mechanism responsible for removing excess cellular cholesterol, particularly from the arterial wall macrophages. Cellular cholesterol removal represents a primary action of HDL against atherosclerosis and depends on its interaction with the ATP-binding cassette transporter A-1 (ABCA-1) and ATP-binding cassette transporter G-1 (ABCG-1) [8] (Figure 2).

Along with facilitating the removal of cellular cholesterol, HDL also mitigates LDL and cell membrane oxidation, as well as the inflammatory response. Characteristically, lipid accumulation predisposes the generation of reactive oxygen species (ROS) and inflammatory signaling pathways, primarily mediated by nuclear factor kappa B (NFKB). The antioxidative and anti-inflammatory effects of HDL are mainly mediated by apoA-1, apoA-4, apoM, and paraoxonase 1 (PON1). Transported by HDL, PON1 plays a key role in neutralizing ROS, regulating vascular tone, inhibiting endothelial adhesion proteins, reducing apoptosis in endothelial cells, and enhancing endothelial integrity repair through hematopoietic precursors. In endothelial and immune cells, HDL impairs the expression of genes involved in inflammation, resulting in reduced secretion of inflammatory cytokines and adhesion molecules involved in vascular damage [9–11].

HDL reorganizes the monocyte cytoskeleton, a step that precedes cell migration in response to a chemotactic stimulus. At the same time, it modifies the phenotypic differentiation and the ability of monocytes to bind to endothelial wall adhesion molecules, limiting their passage into the subendothelial space and thereby preventing the development of atherosclerotic plaque [12, 13]. In the presence of low-intensity inflammation, the role of HDL has been demonstrated in inhibiting the action and migration of monocytes, as evidenced by the lower number of active macrophages in the peritoneal cavity [14].

HDL can improve insulin secretion by altering the cell membrane and the cholesterol content in granules of insulin-secreting cells (B pancreatic cells). Additionally, in peripheral cells, apoA-1 enhances insulin signaling, promoting insulin sensitivity and maintaining glucose homeostasis [15].

More recently, the determination of the proteome and lipidome of HDL has contributed to discerning the differential function of HDL subfractions in various clinical and metabolic contexts. HDL acts as a cargo particle for hundreds of proteins and bioactive lipids involved not only in lipid metabolism but also in the modulation of the complement system, metalloproteinase activity, endothelial function, and hemostasis, among others. By carrying sphingosine 1 phosphate (S1P), HDL can induce endothelial oxide nitric synthase activity and nitric oxide-mediated vasodilation. S1P modulates the acute-phase protein pentraxin 3 (PTX3), alters the differentiation of lymphocyte lineage cells, and reduces dendritic cell activation and cell death, resulting in an anti-inflammatory phenotype [16, 17].

HDL transports small noncoding microRNAs (miRs) capable of blocking the translation of target RNAs. By delivering miRs to target cells via scavenger receptor class B type 1 (SR-B1), HDL participates in the control of gene expression, a mechanism demonstrated to be relevant for atherogenesis and the progression of other chronic and acute diseases [18]. Moreover, HDL modulates innate immunity, a function attributed to its ability to bind bacterial lipopolysaccharides (LPS), thus facilitating their transport to the liver for detoxification rather than binding to Toll-like receptors (TLRs) and triggering inflammatory signaling. This plethora of actions attributed to HDL makes this lipoprotein not strictly linked to CV risk regarding the strict control of lipid homeostasis but also related to the modulation of immune and inflammatory activity, which guides the genesis and evolution of various chronic diseases including the ARDs [12, 19, 20].

## 3. HDLs and ARDs

While the specific treatment of ARDs has advanced considerably in recent years, not all individuals respond satisfactorily, and even those who do respond may not always be able to completely halt the progressive evolution of immune aggression.

A proatherogenic lipid pattern is often observed in ARDs, including elevated levels of plasma low-density lipoprotein cholesterol (LDLc) [21–26]. However, in RA, a reduction in LDLc is observed, which is proportional to the degree of disease activity. This event is described as the lipid paradox in RA, given the higher occurrence of ischemic events in these patients alongside the reduction in LDLc levels. This is attributed to the increase in the fraction of sdLDL, which is more atherogenic due to its greater passage into the arterial intima and susceptibility to oxidative modification, favoring the chemotaxis of immune cells and their uptake by monocyte-derived macrophages [27, 28].

A systematic review showed that circulating levels of vascular cell adhesion molecule 1 (VCAM-1), intercellular adhesion molecule 1 (ICAM-1), and selectin E have a strong correlation with subclinical atherosclerotic disease in RA [29]. The immunological, inflammatory, and oxidative insults accompanying ARDs are key elements in metabolic modulation across different clinical stages of these diseases. Inflammation is one of the mechanisms guiding the genesis of insulin resistance that leads to compensatory hyperinsulinemia and enhanced free fatty acids output from adipose tissue. Consequently, there is increased hepatic production of TG and secretion of VLDL, coupled with reduced pre-beta HDL generation. The reduction in HDLc is driven by the decreased activity of LPL on TG-rich lipoproteins and the increased activity of CETP, which produces larger, TG–enriched HDL particles. These become a better substrate for hepatic lipase, resulting in the formation of smaller HDL particles associated with lower CV protection [30, 31]. Additionally, acute-phase proteins including serum amyloid A (SAA), ceruloplasmin, and haptoglobin, and inflammatory cytokines bind to HDL and displace its main protein component, apoA-1, compromising its ability to mediate RCT and other actions [32]. The increase in phospholipase A2 (PLA2) activity, seen in acute and chronic inflammatory conditions, leads to a reduction in the concentration of PL on the surface of HDL and contributes unfavorably with its actions. Additionally, it produces lysophosphatidylcholine, which intensifies the immune and inflammatory response [31]. In this context, HDL becomes proinflammatory and immunogenic, contributing to a proatherogenic environment alongside other lipid alterations. Indeed, the functional modifications of HDL seem to explain why therapies focused on raising HDLc do not unequivocally correlate with better CV outcomes. This also exacerbates the CV risk in ARDs regardless of disease remission, management with medications, and control of other CV risk factors [7, 33].

PL transfer protein (PLTP) is reported as an inflammatory inducer in subjects with RA, regardless of its role in metabolism. In RA, a preferential deviation of PL from the HDL surface towards the synthesis of arachidonic acid, a precursor of proinflammatory eicosanoids, is observed [34]. Elevated TG levels within HDL particles have been observed in individuals with RA, SLE, and psoriasis, and are associated with a reduction in PL and EC in the HDL [35]. Levels of PLA2 showed a strong association with the presence of subclinical atherosclerotic disease and the intensity of disease activity in individuals with early RA [36].

The capacity of apoB-depleted serum, containing only HDL as lipoprotein, to mediate cholesterol efflux from macrophages (named CEC) was directly related to CV protection in several clinical trials. Furthermore, in most cases, it was shown to be independent of the inflammatory process when adjusted for C-reactive protein (CRP), an important inflammatory biomarker [37]. The predictive power of CEC in acute coronary syndrome was evaluated in the prospective study PREDIMED, with individuals at high CV risk. The relative risk of events was lower with higher CEC, independently of other traditional risk factors, including the absolute values of plasma HDLc [38]. In SLE, there is evidence of a direct relationship between carotid atherosclerotic plaque and CEC [39]. However, in some studies in AR, the data are controversial when trying to correlate atherogenic disease and CEC [40]. Mehta et al. [41] demonstrated a reduction in HDLc levels and lower CEC in psoriasis, along with a greater association of HDL size alterations with signs of CVD. Apo-A1 and apo-M were reduced in HDL in replacement by acute-phase inflammatory proteins [21].

In ARDs, the increase in immune complexes containing fractions of complement C1q reduces the activity of cholesterol 27-hydroxylase in endothelial cells and activated macrophages. This reduces the conversion of cholesterol into 27-hydroxycholesterol, a more soluble oxysterol, which, when exported from cells, contributes as an additional pathway to RCT, mediated by HDL [42].

The expression of the gene that encodes ABCA-1 can be inhibited by inflammatory cytokines such as IL-6, interferon-gamma (IFN-gamma), platelet-derived growth factor (PDGF), and interleukin 1 beta (IL-1beta) or, on the other hand, can be stimulated by interleukin 10 (IL-10) and transforming growth factor beta 1 (TGFbeta1) [43]. This indicates that ABCA-1 is related to the Janus kinase (JAK)/signal transducer and activator of transcription 3 (JAK/STAT3) inflammatory pathway, in which HDL plays an important inhibitory role in addition to its direct role in RCT [12].

In some ARDs, activation of neutrophil myeloperoxidase (MPO) promotes oxidation of apoA-1, compromising LCAT activity and CEC. Additionally, it generates glycolaldehyde, an oxoaldehyde that modifies HDL by advanced glycation, hindering the flow along the RCT and damaging other HDL functions [44, 45].

On the other hand, in RA, an increase in LCAT activity is observed, especially during disease active phase of the disease, alongside a reduction in the ABCA-1-mediated CEC/ABCG-1-mediated CEC ratio, clearly indicating a blockade in the maturation of pre-beta HDL to mature HDL. Systemic inflammation markers such as CRP and RA activity score (DAS28) have an inverse association with ABCA1-dependent CEC. This same spectrum has been observed in healthy volunteers who received intravenous LPS infusion, simulating what is observed in sepsis [46]. In addition, in individuals with RA, impairment of RCT mediated by ABCG-1 was evidenced, correlating with the disease activity inferred by the DAS28 [47].

*Abca1* and *Abcg1* knockout animals in dendritic cells exhibit a SLE-like pattern of adenomegaly and glomerulonephritis, with cellular cholesterol accumulation and activation of proinflammatory pathways, linking to polarization towards T helper lymphocytes 1 (Th1) and Th17, and more M1-inflammatory macrophages. These data suggest that HDL-mediated RCT is important in maintaining immune tolerance [48]. Additionally, in SLE, anti-ABCA-1 antibody titers are higher, proportionally higher in those with atherosclerotic manifestations [49].

The main ARDs, including RA, SLE, AS, PsA, and SS, exhibit quantitative and qualitative alterations in HDL particles [3, 21–24]. Interestingly, despite the observed reduction in the quantity of HDL, a few studies have not shown differences in HDL size compared to control groups, prompting discussions on the possibility of methodological variations. In adolescents with SLE, the HDL-c/apoA-1 ratio was elevated, indicating a lower amount of apoA-1 in the particles and indirectly suggesting the predominance of larger particles [25]. In Behçet's disease, it has been demonstrated that the HDL_2_ fraction is reduced, while HDL_3_ is elevated. The LDLc/HDLc ratio is higher than in controls and is directly related to concentrations of CRP and TG [26]. Plasma HDLc concentration is postulated as predictive of autoimmune disease exacerbation. One study revealed an average 9% decrease in HDLc preceding the onset of symptoms in individuals with RA [50].

In SLE, the low concentration of HDLc involves, besides the reduction in LPL activity, the presence of anti-LPL and anti-apoA-1 antibodies and higher CETP activity. Approximately one-third of individuals with SLE exhibit anti-apoA-1 antibodies, which appear primarily targeted towards mature HDL particles and directly linked to disease activity [51]. Elevated anti-apoA1 antibody titers are correlated with an increase in Systemic Lupus Erythematosus Disease Activity Index (SLEDAI) and are inversely proportional to PON1 activity [52, 53]. These autoantibodies can also be detected in individuals without autoimmune disease, shortly after acute coronary events, although their significance remains poorly understood [54]. Additionally, in SLE, as in RA, HDL particles are less enriched in apoA-1, apoM, and PON1 and have a higher content of SAA [55–57]. McMahon et al. [58] demonstrated in SLE, HDL with proinflammatory characteristics in a higher proportion compared to RA (45% > 20%), and to a control population (4%). However, such comparison is difficult considering the distinct activities of the diseases and the fact that no correlation of these findings was observed in individuals with disease activity in SLE, quantified by the SLEDAI score. Exacerbated immunological responses increase the oxidation of apo A-1, making this apo more immunogenic. This, in turn, further boosts anti-apoA-1 antibody titers and accelerates the degradation of HDL [39, 59].

Parra et al. [60] demonstrated, in a cohort of individuals with SLE, a correlation between small, dense dysfunctional HDL and levels of complement component 3 (C3) and complement component 4 (C4), as well as activation of the complement cascade, in those experiencing acute disease episodes and unfavorable clinical outcomes. The apoM, which anchors S1P, is reduced in patients with SLE, inversely associated with the intensity and activity of the disease, as measured by the SLEDAI score, characterized by leukopenia and elevated titers of anti-dsDNA antibodies and CRP. At the same time, it is positively associated with C3 levels [61].

Interestingly, there are reports of elevated HDLc levels or values within the normal range in cases of ARD, still associated with a higher CV risk [62, 63]. This is described in the general population, with a U-shaped mortality curve for extreme HDLc values. It is important to note that at high HDLc levels above 90 mg/dL, allelic variants in genes encoding SR-B1 and CETP have been identified, which impair the RCT [64–66]. Alteration in HDL functionality is indeed evidenced, with compromised CEC and this alteration is proportional to the degree of ARD activity [42, 48]. Recent observations from our group have demonstrated that elevations in HDLc and HDL composition in PL and total cholesterol, as well as the ability of HDL particles to remove cell cholesterol, reduce the odds ratio for active RA [67].

The content of miR-1246 in HDL is higher in individuals with RA, which is linked to higher IL-6 production, opposing the anti-inflammatory activity of HDL [37]. Moreover, in RA with high inflammatory activity and CRP levels > 10 mg/L, a reduction in the antioxidant capacity of the HDL_3_ subfraction was observed [68].

In some studies, the levels of proinflammatory HDL did not significantly change over time in patients with SLE, at different stages of disease activity, even when treated. This suggests that chronic inflammatory status, albeit of low intensity, is already sufficient to alter HDL, or that such alterations involve genetically predetermined factors or those linked to the etiopathogenesis of autoimmune disease. On the other hand, when individuals with SLE and pre-established CVD were analyzed compared to those without atherosclerosis, levels of proinflammatory HDL were significantly higher in the former group [69].

All aspects discussed explain why traditional CV risk scores underestimate individuals with ARDs, as they fail to account for the inflammatory aspect and its consequences on lipoprotein functionality. Therefore, it would be ideal to incorporate methodologies for quantifying HDL functionality in addition to HDLc in CV risk prediction [70]. Determining HDL functionality encounters methodological challenges that include laborious and costly techniques, which are difficult to compare when considering the different methods of isolating these particles and determining their various actions using different cell types, in vitro assays, and animal models.

The use of recombinant HDL and apoA-1 mimetics is beneficial in reducing the area of atherosclerotic lesions and local inflammatory processes, although there are divergent results [71–73]. The infusion of the apo A-1 mimetic peptide (4FP) reduced morbidity and mortality in animal models of sepsis [74], reinforcing the role of HDL in immune and inflammatory control. Furthermore, in a study using a murine model of autoimmunity, the infusion of apoA-1 could normalize regulatory T lymphocytes (Tregs), which inhibit the activation of other lymphocyte populations, thereby suppressing cell-mediated immune response and inflammatory activity [75].

## 4. Therapeutic Approach in ARDs and HDLs

A more in-depth approach to therapies in ARDs is beyond the scope of this review. However, it is worth considering that various therapies may lead to changes in HDL composition and functionality, and consequently, in the modulation of CV risk in ARDs. Many individuals with active ARDs or joint sequelae end up significantly reducing engagement in physical activities [76]. Regular physical exercise, particularly aerobic activities, raises plasma HDL concentrations. Aerobic exercise for 12-24 weeks increased HDLc by 3.8 to 15.4 mg/dL compared to baseline [30]. Additionally, regular aerobic activity promotes an increase in HDL particle size in apoA-1 content, and greater PON1 activity [76, 77]. Exercise improves lipid profile and also reduces vascular tone, controls blood pressure, and modifies body fat distribution, reducing the waist-to-hip ratio, as demonstrated in individuals with AR [78]. SLE subjects who exercise little have a proinflammatory HDL profile and a higher incidence of subclinical atherosclerosis, as shown in a comparative study with active individuals [79]. In mice, it has been shown that aerobic exercise increases cholesterol trafficking from macrophages to the liver for subsequent biliary excretion through RCT without changes in the expression of genes involved in macrophage cholesterol efflux. In the liver, increased cholesterol uptake from plasma was observed due to the upregulation of SR-B1 and LDL receptors in wild-type mice and transgenic mice expressing human CETP [80].

Statins, which constitute the standard treatment for hypercholesterolemia, are frequently prescribed for individuals with ARDs [81]. The pleiotropic actions of statins extend beyond inhibiting cholesterol synthesis by blocking the enzyme hydroxymethylglutaryl-coenzyme A reductase. They include antioxidant activity, inhibition of metalloproteinases, and stabilization of atherosclerotic plaques. Some studies indicate a modulating effect of statins on lipid microdomains in cell membranes [82]. Statins have been shown to disrupt the binding of oxidized LDL to lectin-like oxidized LDL receptor-1 (LOX-1) on endothelial cells [83] and reduce Toll-like receptors (TLRs), interleukin-2 receptors (IL-2r), and major histocompatibility complex 2 expression [84–87].

In mice, simvastatin stimulated the expression of endothelial SR-B1 through a mechanism involving the peroxisome proliferator–activated receptor alpha (PPAR alpha), which in turn stimulated the endothelial nitric oxide synthase production dependent on HDL and reduced the expression of endothelial adhesion molecules [88]. In another animal model, fluvastatin, through interaction with membrane lipid microdomains, shifted macrophage polarization from M1 to M2, leading to anti-inflammatory, antioxidative, and antiatherogenic effects [89]. Even more compellingly, persistent statin use in a study was associated with a lower risk of developing AR [90]. However, despite these multiple favorable pleiotropic effects of statins, they have not yet demonstrated the ability to reverse the proinflammatory state of HDL, thus leaving residual CV risk [27]. Despite modifying various HDL systems, statins can induce a reduction in HDL cholesterol without exerting a significant effect on CEC [91].

Fibrates as PPAR alpha agonists promote the elevation of HDLc and increase gene transcription of the *ABCA1* gene [92]. Their anti-inflammatory action proves beneficial in the clinical manifestations of RA, with a significant improvement in DAS28 [93, 94].

Although the transient use of corticosteroids has shown an elevation in HDL in RA patients [95], it is known that this therapy can worsen other CV risk factors, compromising the benefits that could result from controlling the inflammatory process [96]. After 2 years of corticosteroid therapy, data analysis revealed an increased risk of CVD in SLE patients, driven by elevated apoB and TG levels, along with reduced HDLc [96]. The adverse effects of corticosteroids are time and dose-dependent, and occasional use during exacerbations to minimize symptoms does not appear to be harmful. On the other hand, chronic doses of prednisone in RA patients result in suppression of CEC and increased progression of atherosclerotic plaque [46].

In general, disease–modifying antirheumatic drugs (DMARDs) improve the lipid profile and enhance the cardioprotective function of HDL [97–99]. Despite some studies reporting contradictory data, a meta-analysis in patients with RA demonstrated that CEC correlates more closely with therapeutic benefit than the absolute quantity of HDL particles [41]. In subjects with early RA, different combinations of DMARDs led to a reduction in disease activity, along with favorable modifications of HDL particles in their structure and functional capacity, quantified by their composition of apoA-1, MPO, and PON1 [100].

In RA, the TNF inhibitor, infliximab, has shown improvement in HDLc, an effect that persisted after 6 months of administration [101]. In general, studies have associated anti-TNF therapies with a reduction of up to 54% in the risk of CV events [102]. Besides the elevation of HDLc, this class is also associated with an increase in LDLc, in total agreement with what was previously described as the lipid paradox of RA, and the effect can also be assessed by the reduction in the apoB/ApoA-1 ratio [103].

Adalimumab in the AMPLE study improved HDL function and CEC and altered HDL proteome, especially structural proteins involved in immune interaction, antioxidant capacity, and anti-inflammatory action. The same profile was observed abatacept, a costimulatory modulating drug that acts on T lymphocytes [104]. Although TNF inhibitors have shown potential to inhibit complement system activation, sustained inhibition was achieved only with the addition of methotrexate in one study, maintained over a 6-month follow-up period [105].

Anti-IL-6 therapies for RA, such as tocilizumab, have been associated with improvements in the atherogenic profile of lipoproteins by modifying HDL in terms of CEC and the expression of SAA, PON1, and PLA2 [106, 107]. A study compared the effects of tocilizumab with adalimumab, and as the actions of IL-6 and TNF are not necessarily similar, the study is aimed at seeing if there were differences in terms of lipid profile. In this regard, tocilizumab was superior in increasing HDLc and LDLc and in reducing HDL-SAA, PLA2, and Lp(a) [108]. In the KALIBRA study, using tocilizumab in RA patients, it was observed that the elevation of LDLc, within the concept of the lipid paradox, resulted from the reduction of LDL catabolism, rather than an increase in the synthesis of this lipoprotein [109].

In a meta-analysis, methotrexate, a first-line drug in the treatment of RA, reduced CV outcomes by 21% [110]. Methotrexate induces the generation and maturation of pre-beta HDL and reduces macrophage differentiation. Moreover, it increased CEC, mediated by ABCG1 and SR-B1 [111]. In a prospective study with 288 subjects with psoriasis, it was observed a proinflammatory lipid profile in those with joint involvement compared to those with exclusive cutaneous manifestation. After the use of methotrexate in PsA, a significant reduction in the apoB/apoA-1 ratio was observed; however, this benefit was observed only in men [112].

Hydroxychloroquine has been shown to increase HDLc in subjects with SLE and RA, improving CEC and reducing non-HDL and TG [113–115]. Chloroquine has also been shown to reduce atherosclerotic outcomes in patients with SLE [116].

In SLE, rituximab reduced plasma TG without a significant alteration in LDLc and HDLc [117]. However, when this drug was administered to individuals with RA, it improved endothelial function and reduced carotid intima-media thickness, despite the lipid profile alteration not being as significant [118]. Another study, however, showed an improvement in the functional and quantitative profile of HDL following the administration of rituximab in subjects with RA [55].

JAK inhibitory therapies increased HDLc in subjects with SLE and RA and raised CEC by enhancing LCAT activity. A meta-analysis with baricitinib, an oral selective JAK1, and JAK2 inhibitor, showed a dose-dependent increase in HDLc [119].

Using the PREDICTS score, which utilizes various clinical and laboratory parameters to quantify the risk of atherosclerosis in SLE, as a method of evaluating the therapeutic response, a significant reduction in the index was obtained with the use of the immunosuppressant mycophenolate mofetil (MMF), but this was not observed with the use of azathioprine or chloroquine This result is noteworthy as elevated PREDICTS scores can increase the risk of atherosclerotic disease by up to 28 times [120]. On the other hand, MMF did not prove to be effective in reducing the progression of subclinical atherosclerosis, as assessed by carotid intima-media thickness and calcium score, although the follow-up period was only 2 years, and the sample size was limited [121]. In an animal model of LDL receptor knockout (LDLr-/-) mouse prone to develop SLE, MMF decreased the size of atherosclerotic lesions and reduced the population of CD4+ T lymphocytes both in the atheromatous lesion and in the periphery, which was not demonstrated in the control group with atorvastatin [122].

Anifrolumab is an inhibitor of interferon 1-mediated signaling, linked to the etiopathogenesis of SLE. In a phase II study (MUSE trial) with SLE patients, its use resulted in a reduction in the formation of neutrophil extracellular traps and TNF concentration and a significant increase in CEC mediated by HDL [123]. Also, in SLE subjects resistant or intolerant to traditional medications, sirolimus, a mammalian target of rapamycin complex 1 (mTORC1) inhibitor, was used, reducing the sensitivity of IL-2r receptors on T and B lymphocytes. A significant improvement in SLEDAI and BILAG activity scores was observed after 12 months of treatment, as well as a reduction in the required corticosteroid dose and an expansion of the Treg population. The only unexpected finding was the maintenance of HDLc plasma levels when an elevation was expected [124].

Knock-out mice for ABCA1 and ABCG1 in dendritic cells show activation of inflammatory pathways and development of ARDs, which are attenuated after the administration of reconstituted HDL (rHDL). Infusion of rHDL was tested in mouse models of RA, which naturally present lower CEC with reduced expression of ABCA1. The therapy inhibited myelopoiesis and prevented the accumulation of monocytes in endothelial lesions [125]. The combination of 4FP with pravastatin was used in experimental models of RA and demonstrated the ability to minimize the inflammatory process and improve the clinical severity score of the disease [126]. Since individuals with the apoA-1_Milano_ allelic variant were shown to be protected against CVD, despite low levels of HDLc, a recombinant mimetic of the same variant (MDCO216) was infused in individuals after acute coronary artery disease, and although significant regression of atherosclerotic plaque was not observed compared to the placebo group, the difference in CEC was 80.4% and 41.6%, respectively, after 5 weeks of follow-up [127]. At the same time, administration of CSL112, a reconstituted plasma-derived apoA-1, was shown to significantly increase CEC in patients with a recent history of acute coronary syndrome [128].

## 5. Conclusions

HDL acts in modulating the inflammatory and immune response through interaction with different cells of the immune system. Therefore, the importance of a more in-depth study of the relationship between HDL and ARDs is justified, to minimize complications resulting from immune aggression and to reduce residual CV risk, despite therapy and control of major traditional risk factors. Although the specific treatment of ARDs has evolved considerably in recent years, not all subjects respond satisfactorily, and those who do respond are not always able to completely halt the progressive evolution of immune aggression. HDL, due to its pleiotropic actions, represents an important effector in the link between ARDs and atherosclerosis.

## Data Availability

Data sharing does not apply to this article as no new data were created or analyzed in this study.

## References

[1] Helmick C. G., Felson D. T., Lawrence R. C. (2008). Estimates of the prevalence of arthritis and other rheumatic conditions in the United States: part I. *Arthritis & Rheumatism*.

[2] Moutsopoulos H. M. (2021). Autoimmune rheumatic diseases: one or many diseases?. *Journal of Translational Autoimmunity*.

[3] Ronda N., Zimetti F., Adorni M. P., Palumbo M., Karpouzas G. A., Bernini F. (2023). Role of lipoprotein levels and function in atherosclerosis associated with autoimmune rheumatic diseases. *Rheumatic Diseases Clinics of North America*.

[4] Ferreira H. B., Pereira A. M., Melo T., Paiva A., Domingues M. R. (2019). Lipidomics in autoimmune diseases with main focus on systemic lupus erythematosus. *Journal of Pharmaceutical and Biomedical Analysis*.

[5] Giles J. T., Danielides S., Szklo M. (2015). Insulin resistance in rheumatoid arthritis: disease-related indicators and associations with the presence and progression of subclinical atherosclerosis. *Arthritis & Rhematology*.

[6] Sánchez-Pérez H., Tejera-Segura B., de Vera-González A. (2017). Insulin resistance in systemic lupus erythematosus patients: contributing factors and relationship with subclinical atherosclerosis. *Clinical and Experimental Rheumatology*.

[7] Misra D. P., Hauge E. M., Crowson C. S., Kitas G. D., Ormseth S. R., Karpouzas G. A. (2023). Atherosclerotic cardiovascular risk stratification in the rheumatic diseases:. *Rheumatic Diseases Clinics of North America*.

[8] Adorni M. P., Ronda N., Bernini F., Zimetti F. (2021). High density lipoprotein cholesterol efflux capacity and atherosclerosis in cardiovascular disease: pathophysiological aspects and pharmacological perspectives. *Cells*.

[9] Barter P. J., Nicholls S., Rye K. A., Anantharamaiah G. M., Navab M., Fogelman A. M. (2004). Anti-inflammatory properties of HDL. *Circulation Research*.

[10] Riwanto M., Landmesser U. (2013). High density lipoproteins and endothelial functions: mechanistic insights and alterations in cardiovascular disease. *Journal of Lipid Research*.

[11] Rozenberg O., Rosenblat M., Coleman R., Shih D. M., Aviram M. (2003). Paraoxonase (PON1) deficiency is associated with increased macrophage oxidative stress: studies in PON1-knockout mice. *Free Radical Biology & Medicine*.

[12] Trakaki A., Marsche G. (2021). Current understanding of the immunomodulatory activities of high-density lipoproteins. *Biomedicine*.

[13] Murphy A. J., Westerterp M., Yvan-Charvet L., Tall A. R. (2012). Anti-atherogenic mechanisms of high density lipoprotein: effects on myeloid cells. *Biochimica et Biophysica Acta (BBA)-Molecular and Cell Biology of Lipids*.

[14] McGillicuddy F. C., de la Llera M. M., Hinkle C. C. (2009). Inflammation impairs reverse cholesterol transport in vivo. *Circulation*.

[15] King T. W., Cochran B. J., Rye K.-A. (2023). ApoA-I and diabetes. *Arteriosclerosis, Thrombosis, and Vascular Biology*.

[16] Liu G., Yang K., Burns S., Shrestha S., Chi H. (2010). The S1P_1_-mTOR axis directs the reciprocal differentiation of T_H_1 and T_reg_ cells. *Nature Immunology*.

[17] Blaho V. A., Hla T. (2014). An update on the biology of sphingosine 1-phosphate receptors. *Journal of Lipid Research*.

[18] Kajani S., Curley S., McGillicuddy F. C. (2018). Unravelling HDL–looking beyond the cholesterol surface to the quality within. *International Journal of Molecular Sciences*.

[19] Beck W. H., Adams C. P., Biglang-Awa I. M. (2013). Apolipoprotein A–I binding to anionic vesicles and lipopolysaccharides: role for lysine residues in antimicrobial properties. *Biochimica et Biophysica Acta (BBA)-Biomembranes*.

[20] Suzuki M., Pritchard D. K., Becker L. (2010). High-density lipoprotein suppresses the type I interferon response, a family of potent antiviral immunoregulators, in macrophages challenged with lipopolysaccharide. *Circulation*.

[21] Holzer M., Wolf P., Curcic S. (2012). Psoriasis alters HDL composition and cholesterol efflux capacity. *Journal of Lipid Research*.

[22] Gkolfinopoulou C., Stratikos E., Theofilatos D. (2015). Impaired antiatherogenic functions of high-density lipoprotein in patients with ankylosing spondylitis. *The Journal of Rheumatology*.

[23] Ferraz-Amaro I., Hernández-Hernández M. V., Armas-González E., Sánchez-Pérez H., Machado J. D., Díaz-González F. (2020). HDL cholesterol efflux capacity is related to disease activity in psoriatic arthritis patients. *Clinical Rheumatology*.

[24] Rodríguez-Carrio J., Mozo L., López P., Nikiphorou E., Suárez A. (2018). Anti-high-density lipoprotein antibodies and antioxidant dysfunction in immune-driven diseases. *Frontiers in Medicine*.

[25] Machado D., Sarni R. O., Abad T. T. (2017). Lipid profile among girls with systemic lupus erythematosus. *Rheumatology International*.

[26] Messedi M., Jamoussi K., Frigui M. (2011). Atherogenic lipid profile in Behçet’s disease: evidence of alteration of HDL subclasses. *Archives of Medical Research*.

[27] Gervois P., Torra I. P., Fruchart J. C., Staels B. (2000). Regulation of lipid and lipoprotein metabolism by PPAR activators. *Clinical Chemistry and Laboratory Medicine*.

[28] Myasoedova E., Crowson C. S., Kremers H. M. (2011). Lipid paradox in rheumatoid arthritis: the impact of serum lipid measures and systemic inflammation on the risk of cardiovascular disease. *Annals of the Rheumatic Diseases*.

[29] Mangoni A. A., Zinellu A. (2024). A systematic review and meta-analysis of circulating adhesion molecules in rheumatoid arthritis. *Inflammation Research*.

[30] Chu K.-H. (2022). The current status of research on high-density lipoproteins (HDL): a paradigm shift from HDL quantity to HDL quality and HDL functionality. *International Journal of Molecular Sciences*.

[31] Bonacina F., Pirillo A., Catapano A., Norata G. D. (2021). HDL in immune-inflammatory responses: implications beyond cardiovascular diseases. *Cells*.

[32] Van Lenten B. J., Hama S. Y., de Beer F. C. (1995). Anti-inflammatory HDL becomes pro-inflammatory during the acute phase response. Loss of protective effect of HDL against LDL oxidation in aortic wall cell cocultures. *The Journal of Clinical Investigation*.

[33] Xiang A. S., Kingwell B. A. (2019). Rethinking good cholesterol: a clinician’s guide to understanding HDL. *The Lancet Diabetes and Endocrinology*.

[34] Giraud C., Tournadre A., Pereira B. (2019). Alterations of HDL particle phospholipid composition and role of inflammation in rheumatoid arthritis. *Journal of Physiology and Biochemistry*.

[35] Kontush A., Lhomme M., Chapman M. J. (2013). Unraveling the complexities of the HDL lipidome. *Journal of Lipid Research*.

[36] Södergren A., Karp K., Bengtsson C., Möller B., Rantapää-Dahlqvist S., Wållberg-Jonsson S. (2015). Is lipoprotein-associated phospholipase A2 a link between inflammation and subclinical atherosclerosis in rheumatoid arthritis?. *BioMed Research International*.

[37] Wu Q., Sheng Q., Michell D. (2024). Anti-inflammatory effect ofhigh‐densitylipoprotein blunted by delivery of alteredmicroRNAcargo in patients with rheumatoid arthritis. *Arthritis & Rhematology*.

[38] Soria-Florido M. T., Castañer O., Lassale C. (2020). Dysfunctional high-density lipoproteins are associated with a greater incidence of acute coronary syndrome in a population at high cardiovascular risk. *Circulation*.

[39] Kim S. Y., Yu M., Morin E. E., Kang J., Kaplan M. J., Schwendeman A. (2020). High-density lipoprotein in lupus: disease biomarkers and potential therapeutic strategy. *Arthritis and Rheumatism*.

[40] Xie B., He J., Liu Y., Liu T., Liu C. (2021). A meta-analysis of HDL cholesterol efflux capacity and concentration in patients with rheumatoid arthritis. *Lipids in Health and Disease*.

[41] Mehta N. N., Li R., Krishnamoorthy P. (2012). Abnormal lipoprotein particles and cholesterol efflux capacity in patients with psoriasis. *Atherosclerosis*.

[42] Reiss A. B., Awadallah N. W., Malhotra S. (2001). Immune complexes and IFN-*γ* decrease cholesterol 27-hydroxylase in human arterial endothelium and macrophages. *Journal of Lipid Research*.

[43] Yin K., Liao D. F., Tang C. K. (2010). ATP-binding membrane cassette transporter A1 (ABCA1): a possible link between inflammation and reverse cholesterol transport. *Molecular Medicine*.

[44] Vivekanandan-Giri A., Slocum J. L., Byun J. (2013). High density lipoprotein is targeted for oxidation by myeloperoxidase in rheumatoid arthritis. *Annals of the Rheumatic Diseases*.

[45] Alisik T., Alisik M., Nacir B., Ayhan F. F., Genc H., Erel O. (2021). Evaluation of dysfunctional high-density lipoprotein levels with myeloperoxidase/paraoxonase-1 ratio in rheumatoid arthritis. *International Journal of Clinical Practice*.

[46] Karpouzas G. A., Papotti B., Ormseth S. R. (2023). Inflammation and immunomodulatory therapies influence the relationship between ATP-binding cassette A1 membrane transporter-mediated cholesterol efflux capacity and coronary atherosclerosis in rheumatoid arthritis. *Journal of Translational Autoimmunity*.

[47] Ronda N., Favari E., Borghi M. O. (2014). Impaired serum cholesterol efflux capacity in rheumatoid arthritis and systemic lupus erythematosus. *Annals of the Rheumatic Diseases*.

[48] Westerterp M., Gautier E. L., Ganda A. (2017). Cholesterol accumulation in dendritic cells links the inflammasome to acquired immunity. *Cell Metabolism*.

[49] Zeng T., Li S. J., Ao W. (2012). The detection of autoantibodies to ATP-binding cassette transporter A1 and its role in the pathogenesis of atherosclerosis in patients with systemic lupus erythematosus. *Clinical Biochemistry*.

[50] Nurmohamed M. T. (2007). Atherogenic lipid profiles and its management in patients with rheumatoid arthritis. *Vascular Health and Risk Management*.

[51] Dinu A. R., Merrill J. T., Shen C., Antonov I. V., Myones B. L., Lahita R. G. (1998). Frequency of antibodies to the cholesterol transport protein apolipoprotein A1 in patients with SLE. *Lupus*.

[52] Kiss E., Seres I., Tarr T., Kocsis Z., Szegedi G., Paragh G. (2007). Reduced paraoxonase1 activity is a risk for atherosclerosis in patients with systemic lupus erythematosus. *Annals of the New York Academy of Sciences*.

[53] Batuca J. R., Ames P. R. J., Isenberg D. A., Delgado A. J. (2007). Antibodies toward high-density lipoprotein components inhibit paraoxonase activity in patients with systemic lupus erythematosus. *Annals of the New York Academy of Sciences*.

[54] Carbone F., Nencioni A., Mach F., Vuilleumier N., Montecucco F. (2013). Evidence on the pathogenic role of auto-antibodies in acute cardiovascular diseases. *Thrombosis and Haemostasis*.

[55] Raterman H. G., Levels H., Voskuyl A. E., Lems W. F., Dijkmans B. A., Nurmohamed M. T. (2013). HDL protein composition alters from proatherogenic into less atherogenic and proinflammatory in rheumatoid arthritis patients responding to rituximab. *Annals of the Rheumatic Diseases*.

[56] McMahon M., Grossman J., Skaggs B. (2009). Dysfunctional proinflammatory high‐density lipoproteins confer increased risk of atherosclerosis in women with systemic lupus erythematosus. *Arthritis and Rheumatism*.

[57] Du W., Shen T., Li H. (2017). Low apolipoprotein M serum levels correlate with systemic lupus erythematosus disease activity and apolipoprotein M gene polymorphisms with lupus. *Lipids in Health and Disease*.

[58] McMahon M., Grossman J., FitzGerald J. (2006). Proinflammatory high‐density lipoprotein as a biomarker for atherosclerosis in patients with systemic lupus erythematosus and rheumatoid arthritis. *Arthritis and Rheumatism*.

[59] Srivastava R., Yu S., Parks B. W., Black L. L., Kabarowski J. H. (2011). Autoimmune-mediated reduction of high-density lipoprotein cholesterol and paraoxonase 1 activity in systemic lupus erythematosus prone gld mice. *Arthritis & Rhematology*.

[60] Parra S., Vives G., Ferré R. (2012). Complement system and small HDL particles are associated with subclinical atherosclerosis in SLE patients. *Atherosclerosis*.

[61] Tydén H., Lood C., Jönsen A. (2019). Low plasma concentrations of apolipoprotein M are associated with disease activity and endothelial dysfunction in systemic lupus erythematosus. *Arthritis Research & Therapy*.

[62] Cure E., Icli A., Uslu A. U. (2018). Atherogenic index of plasma: a useful marker for subclinical atherosclerosis in ankylosing spondylitis: AIP associate with cIMT in AS. *Clinical Rheumatology*.

[63] Gonzáles-Gay M. A., González-Jaunty C. (2014). Inflammation and lipid profile in rheumatoid arthritis: bridging an apparent paradox. *Annals of the Rheumatic Diseases*.

[64] Madsen C. M., Varbo A., Nordestgaard B. G. (2017). Extreme high high-density lipoprotein cholesterol is paradoxically associated with high mortality in men and women: two prospective cohort studies. *European Heart Journal*.

[65] Madsen C. M., Varbo A., Nordestgaard B. G. (2019). Low HDL cholesterol and high risk of autoimmune disease: two population-based cohort studies including 117341 individuals. *Clinical Chemistry*.

[66] Barter P. J., Caulfield M., Eriksson M. (2007). Effects of torcetrapib in patients at high risk for coronary events. *The New England Journal of Medicine*.

[67] Giacaglia M. B., Felix V. P., Santana M. F. M. (2024). The composition of the HDL particle and its capacity to remove cellular cholesterol are associated with a reduced risk of developing active inflammatory rheumatoid arthritis. *International Journal of Molecular Sciences*.

[68] Gómez Rosso L., Lhomme M., Meroño T. (2014). Altered lipidome and antioxidative activity of small, dense HDL in normolipidemic rheumatoid arthritis: relevance of inflammation. *Atherosclerosis*.

[69] Hahn B. H., Grossman J., Chen W., McMahon M. (2007). The pathogenesis of atherosclerosis in autoimmune rheumatic diseases: roles of inflammation and dyslipidemia. *Journal of Autoimmunity*.

[70] Choy E., Ganeshalingam K., Semb A. G., Szekanecz Z., Nurmohamed M. (2014). Cardiovascular risk in rheumatoid arthritis: recent advances in the understanding of the pivotal role of inflammation, risk predictors and the impact of treatment. *Rheumatology*.

[71] Nicholls S. J., Andrews J., Kastelein J. J. P. (2018). Effect of serial infusions of CER-001, a pre-*β* high-density lipoprotein mimetic, on coronary atherosclerosis in patients following acute coronary syndromes in the CER-001 atherosclerosis regression acute coronary syndrome trial: a randomized clinical trial. *JAMA Cardiology*.

[72] Murphy A. J., Woollard K. J., Suhartoyo A. (2011). Neutrophil activation is attenuated by high-density lipoprotein and apolipoprotein A-I in in vitro and in vivo models of inflammation. *Arteriosclerosis, Thrombosis, and Vascular Biology*.

[73] Kootte R. S., Smits L. P., van der Valk F. M. (2015). Effect of open-label infusion of an ApoA-I-containing particle (CER-001) on RCT and artery wall thickness in patients with FHA. *Journal of Lipid Research*.

[74] Dai L., Datta G., Zhang Z. (2010). The apolipoprotein A-I mimetic peptide 4F prevents defects in vascular function in endotoxemic rats. *Journal of Lipid Research*.

[75] Hyka N., Dayer J. M., Modoux C. (2001). Apolipoprotein AI inhibits the production of interleukin-1*β* and tumor necrosis factor-*α* by blocking contact-mediated activation of monocytes by T lymphocytes. *Blood*.

[76] AbouAssi H., Connelly M. A., Bateman L. A. (2017). Does a lack of physical activity explain the rheumatoid arthritis lipid profile?. *Lipids in Health and Disease*.

[77] Lee H., Park J. E., Choi I., Cho K. H. (2009). Enhanced functional and structural properties of high-density lipoproteins from runners and wrestlers compared to throwers and lifters. *BMB Reports*.

[78] Byram K. W., Oeser A. M., Linton M. F., Fazio S., Stein C. M., Ormseth M. J. (2018). Exercise is associated with increased small HDL particle concentration and decreased vascular stiffness in rheumatoid arthritis. *JCR: Journal of Clinical Rheumatology*.

[79] Volkmann E. R., Grossman J. M., Sahakian L. J. (2010). Low physical activity is associated with proinflammatory high-density lipoprotein and increased subclinical atherosclerosis in women with systemic lupus erythematosus. *Arthritis Care & Research: Official Journal of the American College of Rheumatology*.

[80] Pinto P. R., Rocco D. D., Okuda L. S. (2015). Aerobic exercise training enhances the in vivo cholesterol trafficking from macrophages to the liver independently of changes in the expression of genes involved in lipid flux in macrophages and aorta. *Lipids in Health and Disease*.

[81] Jorge A. M., Lu N., Keller S. F., Rai S. K., Zhang Y., Choi H. K. (2018). The effect of statin use on mortality in systemic autoimmune rheumatic diseases. *The Journal of Rheumatology*.

[82] Kirsch C., Eckert G. P., Mueller W. E. (2003). Statin effects on cholesterol micro-domains in brain plasma membranes. *Biochemical Pharmacology*.

[83] Matarazzo S., Quitadamo M. C., Mango R., Ciccone S., Novelli G., Biocca S. (2012). Cholesterol-lowering drugs inhibit lectin-like oxidized low-density lipoprotein-1 receptor function by membrane raft disruption. *Molecular Pharmacology*.

[84] Chansrichavala P., Chantharaksri U., Sritara P., Ngaosuwankul N., Chaiyaroj S. C. (2010). Atorvastatin affects TLR4 clustering via lipid raft modulation. *International Immunopharmacology*.

[85] Goebel J., Logan B., Forrest K., Mieczkowski A., Roszman T. L., Wills-Karp M. (2005). Atorvastatin affects interleukin-2 signaling by altering the lipid raft enrichment of the interleukin-2 receptor beta chain. *Journal of Investigative Medicine*.

[86] Wei Y. M., Li X., Xiong J. (2013). Attenuation by statins of membrane raft-redox signaling in coronary arterial endothelium. *The Journal of Pharmacology and Experimental Therapeutics*.

[87] Ghittoni R., Napolitani G., Benati D. (2006). Simvastatin inhibits the MHC class II pathway of antigen presentation by impairing Ras superfamily GTPases. *European Journal of Immunology*.

[88] Kimura T., Mogi C., Tomura H. (2008). Induction of scavenger receptor class B type I is critical for simvastatin enhancement of high-density lipoprotein-induced anti-inflammatory actions in endothelial cells. *Journal of Immunology*.

[89] Kauerova S., Bartuskova H., Muffova B. (2021). Statins directly influence the polarization of adipose tissue macrophages: a role in chronic inflammation. *Biomedicines*.

[90] Chodick G., Amital H., Shalem Y. (2010). Persistence with statins and onset of rheumatoid arthritis: a population-based cohort study. *PLoS Medicine*.

[91] Gomaraschi M., Adorni M. P., Banach M., Bernini F., Franceschini G., Calabresi L. (2015). Effects of established hypolipidemic drugs on HDL concentration, subclass distribution, and function. *Handbook of Experimental Pharmacology*.

[92] Ogata M., Tsujita M., Hossain M. A. (2009). On the mechanism for PPAR agonists to enhance ABCA1 gene expression. *Atherosclerosis*.

[93] Shirinsky I., Polovnikova O., Kalinovskaya N., Shirinsky V. (2013). The effects of fenofibrate on inflammation and cardiovascular markers in patients with active rheumatoid arthritis: a pilot study. *Rheumatology International*.

[94] van Eekeren I. C., Clockaerts S., Bastiaansen-Jenniskens Y. M. (2013). Fibrates as therapy for osteoarthritis and rheumatoid arthritis? A systematic review. *Therapeutic Advances in Musculoskeletal Disease*.

[95] García-Gómez C., Nolla J. M., Valverde J., Narváez J., Corbella E., Pintó X. (2008). High HDL-cholesterol in women with rheumatoid arthritis on low-dose glucocorticoid therapy. *European Journal of Clinical Investigation*.

[96] Karp I., Abrahamowicz M., Fortin P. R. (2008). Recent corticosteroid use and recent disease activity: independent determinants of coronary heart disease risk factors in systemic lupus erythematosus?. *Arthritis and Rheumatism*.

[97] Naerr G. W., Rein P., Saely C. H., Drexel H. (2016). Effects of synthetic and biological disease modifying antirheumatic drugs on lipid and lipoprotein parameters in patients with rheumatoid arthritis. *Vascular Pharmacology*.

[98] Charles-Schoeman C., Gonzalez-Gay M. A., Kaplan I. (2016). Effects of tofacitinib and other DMARDs on lipid profiles in rheumatoid arthritis: implications for the rheumatologist. *Seminars in Arthritis and Rheumatism*.

[99] Cacciapaglia F., Perniola S., Venerito V. (2022). The impact of biologic drugs on high-density lipoprotein cholesterol efflux capacity in rheumatoid arthritis patients. *Journal of Clinical Rheumatology*.

[100] Charles-Schoeman C., Yin Lee Y., Shahbazian A. (2017). Improvement of high‐density lipoprotein function in patients with early rheumatoid arthritis treated with methotrexate monotherapy or combination therapies in a randomized controlled trial. *Arthritis & Rhematology*.

[101] Popa C., van Tits L. J., Barrera P. (2009). Anti-inflammatory therapy with tumour necrosis factor alpha inhibitors improves high-density lipoprotein cholesterol antioxidative capacity in rheumatoid arthritis patients. *Annals of the Rheumatic Diseases*.

[102] Barnabe C., Martin B. J., Ghali W. A. (2011). Systematic review and meta-analysis: anti–tumor necrosis factor *α* therapy and cardiovascular events in rheumatoid arthritis. *Arthritis Care and Research*.

[103] Daïen C. I., Duny Y., Barnetche T., Daurès J. P., Combe B., Morel J. (2012). Effect of TNF inhibitors on lipid profile in rheumatoid arthritis: a systematic review with meta-analysis. *Annals of the Rheumatic Diseases*.

[104] Charles-Schoeman C., Gugiu G. B., Ge H. (2018). Remodeling of the HDL proteome with treatment response to abatacept or adalimumab in the AMPLE trial of patients with rheumatoid arthritis. *Atherosclerosis*.

[105] Nguyen T. H. P., Hokstad I., Fagerland M. W. (2022). Antirheumatic therapy is associated with reduced complement activation in rheumatoid arthritis. *PLoS One*.

[106] Pierini F. S., Botta E., Soriano E. R. (2021). Effect of tocilizumab on LDL and HDL characteristics in patients with rheumatoid arthritis. An observational study. *Rheumatology and Therapy*.

[107] McInnes I. B., Thompson L., Giles J. T. (2015). Effect of interleukin-6 receptor blockade on surrogates of vascular risk in rheumatoid arthritis: measure, a randomised, placebo-controlled study. *Annals of the Rheumatic Diseases*.

[108] Gabay C., McInnes I. B., Kavanaugh A. (2016). Comparison of lipid and lipid-associated cardiovascular risk marker changes after treatment with tocilizumab or adalimumab in patients with rheumatoid arthritis. *Annals of the Rheumatic Diseases*.

[109] Robertson J., Porter D., Sattar N. (2017). Interleukin-6 blockade raises LDL via reduced catabolism rather than via increased synthesis: a cytokine-specific mechanism for cholesterol changes in rheumatoid arthritis. *Annals of the Rheumatic Diseases*.

[110] Micha R., Imamura F., Wyler von Ballmoos M. (2011). Systematic review and meta-analysis of methotrexate use and risk of cardiovascular disease. *The American Journal of Cardiology*.

[111] Ronda N., Greco D., Adorni M. P. (2015). Newly identified antiatherosclerotic activity of methotrexate and adalimumab: complementary effects on lipoprotein function and macrophage cholesterol metabolism. *Arthritis & Rhematology*.

[112] Wang B., Deng H., Hu Y. (2022). The difference of lipid profiles between psoriasis with arthritis and psoriasis without arthritis and sex-specific downregulation of methotrexate on the apolipoprotein B/apolipoprotein A-1 ratio. *Arthritis Research & Therapy*.

[113] Morris S. J., Wasko M. C., Antohe J. L. (2011). Hydroxychloroquine use associated with improvement in lipid profiles in rheumatoid arthritis patients. *Arthritis Care and Research*.

[114] Lang M. G., Vinagre C. G., Bonfa E. (2022). Hydroxychloroquine increased cholesterol transfer to high-density lipoprotein in systemic lupus erythematosus: a possible mechanism for the reversal of atherosclerosis in the disease. *Lupus*.

[115] Rempenault C., Combe B., Barnetche T. (2018). Metabolic and cardiovascular benefits of hydroxychloroquine in patients with rheumatoid arthritis: a systematic review and meta-analysis. *Annals of the Rheumatic Diseases*.

[116] Fessler B. J., Alarcón G. S., Jr M. G. G. (2005). Systemic lupus erythematosus in three ethnic groups: XVI. Association of hydroxychloroquine use with reduced risk of damage accrual. *Arthritis & Rheumatism*.

[117] Fernández-Nebro A., Marenco J. L., López-Longo F. (2014). The effects of rituximab on the lipid profile of patients with active systemic lupus erythematosus: results from a nationwide cohort in Spain (LESIMAB). *Lupus*.

[118] Kerekes G., Soltész P., Dér H. (2009). Effects of rituximab treatment on endothelial dysfunction, carotid atherosclerosis, and lipid profile in rheumatoid arthritis. *Clinical Rheumatology*.

[119] Qiu C., Zhao X., She L. (2019). Baricitinib induces LDL-C and HDL-C increases in rheumatoid arthritis: a meta-analysis of randomized controlled trials. *Lipids in Health and Disease*.

[120] McMahon M., Skaggs B., Grossman J. (2019). Comparison of PREDICTS atherosclerosis biomarker changes after initiation of new treatments in patients with SLE. *Lupus Science & Medicine*.

[121] Kiani A. N., Magder L. S., Petri M. (2012). Mycophenolate mofetil (MMF) does not slow the progression of subclinical atherosclerosis in SLE over 2 years. *Rheumatology International*.

[122] van Leuven S. I., Mendez-Fernandez Y. V., Wilhelm A. J. (2012). Mycophenolate mofetil but not atorvastatin attenuates atherosclerosis in lupus-prone LDLr(-/-) mice. *Annals of the Rheumatic Diseases*.

[123] Casey K. A., Smith M. A., Sinibaldi D. (2021). Modulation of cardiometabolic disease markers by type I interferon inhibition in systemic lupus erythematosus. *Arthritis & Rhematology*.

[124] Lai Z. W., Kelly R., Winans T. (2018). Sirolimus in patients with clinically active systemic lupus erythematosus resistant to, or intolerant of, conventional medications: a single-arm, open-label, phase 1/2 trial. *Lancet*.

[125] Dragoljevic D., Kraakman M. J., Nagareddy P. R. (2018). Defective cholesterol metabolism in haematopoietic stem cells promotes monocyte-driven atherosclerosis in rheumatoid arthritis. *European Heart Journal*.

[126] Charles-Schoeman C., Banquerigo M. L., Hama S. (2008). Treatment with an apolipoprotein A-1 mimetic peptide in combination with pravastatin inhibits collagen-induced arthritis. *Clinical Immunology*.

[127] Nicholls S. J., Puri R., Ballantyne C. M. (2018). Effect of infusion of high-density lipoprotein mimetic containing recombinant apolipoprotein A-I Milano on coronary disease in patients with an acute coronary syndrome in the MILANO-PILOT trial: a randomized clinical trial. *JAMA Cardiology*.

[128] Michael Gibson C., Korjian S., Tricoci P. (2016). Safety and tolerability of CSL112, a reconstituted, infusible, plasma-derived apolipoprotein AI, after acute myocardial infarction: the AEGIS-I trial (ApoA-I event reducing in ischemic syndromes I). *Circulation*.

